# A Six-Year-Old Child With Mosaic Trisomy 13

**DOI:** 10.7759/cureus.18346

**Published:** 2021-09-28

**Authors:** Rawia F Albar, Mohammed S Alghamdi, Ahmed M Almasrahi, Mohammed K Aldawsari, Faisal F Aljahdali, Abdulrhman S Alhwaity

**Affiliations:** 1 General Pediatrics, King Abdulaziz Medical City, Jeddah, SAU; 2 Pediatrics, King Saud Bin Abdulaziz University for Health Sciences College of Medicine, Jeddah, SAU

**Keywords:** trisomy 13, patau syndrome, developmental delay, polydactyly, genetic disorder

## Abstract

Trisomy 13 was first described by Patau in 1960. It is a rare genetic disease caused by having an extra copy of chromosome 13. Mosaic trisomy 13 happens when a percentage of the cells are trisomic for chromosome 13, while the remaining cells are euploid. Patau syndrome has a limited survival rate, and most of the carriers die before completing the first year of life. Unlike Patau syndrome, mosaic trisomy 13 is known for longer survival. It is associated with central nervous system malformations, cardiac defects, and psychomotor delay. We report a six-year-old male patient, the third child of a first-degree consanguinity. Born at term via emergency cesarean section due to meconium-stained amniotic fluid and fetal distress. Apgar score nine at one minute and nine at five minutes. Initial examination showed typical dysmorphic features like deep-seated eyes, small palpebral fissure, low set of ears, high arched palate, short neck, and right-hand polydactyly. The diagnosis was made through chromosomal analysis, and it revealed mosaic trisomy 13.

## Introduction

Trisomy 13, or Patau syndrome, is a rare genetic disease. It is due to having an additional copy of chromosome 13. The prevalence of trisomy 13 is estimated to be between 1 in 12000 and 1 in 29000 in live births. Unfortunately, there is no data in the literature about the prevalence of trisomy 13 in Saudi Arabia. Trisomy 13 is known for a limited survival rate, around 28% of the infants die by the first week and 44% of them die by the first month of life. Moreover, the majority of them, around 86%, die by the first year. Survival after the first year is rare, and there are few other reported cases that survived beyond 10 years [1]. Trisomy 13 is associated with central nervous system malformations like holoprosencephaly, congenital heart defects like atrial-septal defect and ventricular-septal defect, psychomotor delay, and mental retardation [2]. Early death is mainly attributed to central nervous system and cardiac anomalies [3]. In this case report, we described a patient with mosaic trisomy 13 who was born in 2015 and is still alive.

## Case presentation

The patient is a six-year-old male, the third child in the family with a positive history of a first-degree consanguinity. The other two siblings are completely healthy and there is no family history of genetic disease. The mother is healthy and became pregnant at age 23. There were no complications during the pregnancy. She was positive for group B streptococcus and was adequately treated with antibiotics. The child is a product of full-term pregnancy, delivered through emergent cesarean section due to meconium-stained amniotic fluid and fetal distress. His Apgar score was nine at one minute and nine at five minutes. 3.1 kg (25th percentile), 49 cm (25th percentile), and 34 cm (25th percentile) are the weight, length, and head circumference measurements at birth, respectively. On initial examination, it showed dysmorphic features like deep-seated eyes, small palpebral fissure, low set of ears, high arched palate, short neck, and right-hand polydactyly. The patient was hypertonic on all four limbs. At 12 hours of life, precordial examination revealed a grade 2/6 systolic ejection murmur at the left lower sternal border. On pulmonary auscultation, he was ventilated on Assist-Control mode with equal bilateral air entry and no pathological sounds. Abdominal examination revealed umbilical hernia which resolved at seven months of age without any intervention, and normal male genitalia with both testicles present in the scrotum. Chromosomal analysis performed on a cultured peripheral blood sample revealed a mosaic result. Five cells showed an additional chromosome 13 (47, XY, +13). Another five cells showed numerical and structural normal male chromosomes (46, XY).

The patient stayed in the neonatal intensive care unit (NICU) for a month because of persistent hyperinsulinemic hypoglycemia, pericardial effusion, and respiratory distress. He was treated with polycose and diazoxide, and the goal was to maintain blood glucose level as 5-6 mmol/L. The pericardial effusion resolved on its own and did not need pericardiocentesis. Intubation was performed for respiratory distress because of worsening oxygen saturation. During his stay in the NICU, he was followed up by endocrinology and cardiology. Head and abdominal ultrasounds were normal and chest X-ray showed cardiomegaly. Echocardiogram showed patent foramen ovale and patent ductus arteriosus, both closed by themselves. Patent ductus arteriosus closed few days after birth and the patent foramen ovale closed at the age of nine months. MRI of the brain done on the 12th day of life showed no evidence of structural malformation and no masses or collections. At 15 months of age, he was referred to plastic surgery for removal of the postaxial polydactyly in the right hand.

The patient is currently being followed by a pediatrician for his asthma which is under control. At the age of 12 months, he presented to the emergency room (ER) for bronchopneumonia with hyperactive airway disease exacerbation. His hyperactive airway disease was controlled since. He is on salbutamol, taken as needed, and fluticasone 50 mcg twice daily with no day or night symptoms, no exercise-induced symptoms, and no ER visits. He used to follow with endocrinology for transient congenital hyperinsulinemia and he was on diazoxide. It resolved at the age of 18 months. Currently, he is being followed by otolaryngology because of snoring. Prescribed mometasone with the possibility of surgical removal of adenoid hypertrophy if not improved with medication.

At the age of two months, he was active, moving all four limbs, mildly holding his head. At age of seven months he was able to sit by himself. At 12 months he was able to cruise around furniture, holds a bottle, and say mama and dada. He was referred to physiotherapy because of balance issues when standing and walking, he showed poor knee control with partial flexion and bowing. According to the mother, his balance issues improved with physiotherapy sessions. At 15 months, he was able to get objects from the floor while standing without losing control and walk a long distance with no support. At 25 months, he was able to say multiple words but unable to form a sentence, he could run and climb up and down the stairs with on and off-balance issues. At four years and five months, he began care with developmental pediatric clinic. They have noticed that his motor activity improved with physical therapy, and he still used single words and no phrases, did not know body parts, was good with non-verbal expression, responds to name, and follows commands. He was noted to be a risk-taker with no sense of danger. The developmental pediatrician assessed his speech and motor skills to be at the level of a two-year-old child. At the age of six years, he continued to make slow progress and he gained new skills like using two to three words phrases and knows some colors and numbers. He is still not trained to use the toilet. The parents were advised to enrol their son in different special education centers to help with his developmental delay.

## Discussion

Trisomy 13 was first described by Patau in 1960. 1/12000 to 1/29000 is the incidence rate seen in live births. It most commonly results in spontaneous abortions. It happens due to the non-disjunction of the germ cells of either parent, and it can occur in meiosis I or II. However, non-disjunction of the maternal germ cell leads to 91% of the cases [4]. Mosaic trisomy 13 is reported in 6% of individuals with trisomy 13, and it is characterized by Robertsonian translocation [4-5]. In mosaicism, a combination of both trisomy 13 and euploid cells are present in variable percentages [5]. Trisomy 13 is most of the time not inherited, and it occurs due to spontaneous events affecting the cell division. If it is inherited, it can be due to balanced translocation [6]. The definitive diagnosis of trisomy 13 is made through chromosomal analysis [4].

Trisomy 13 is associated with severe central nervous system malformations, psychomotor retardation, cardiovascular defects, genitourinary and ocular malformations. Most of the patients with trisomy 13 die before completing the first year of life and only 5%-10% can survive beyond the first year. In contrast to complete trisomy 13, patients with mosaic trisomy 13 present with fewer and milder anomalies and may survive to late childhood and adulthood [7]. The diagnosis in this case report was made through chromosomal analysis of cultured peripheral blood sample, and it revealed a mosaic result. Five cells showed an additional chromosome 13 (47, XY, +13), and another five cells showed normal male chromosomes (46, XY).

Mosaic trisomy 13 patients may show improvement in motor and mental deficits as seen in our case [8]. Morán-Barroso et al. reported a 12-year-old female patient with mosaic proximal trisomy 13. Comparing with our patient, they showed different development. They reported that she could support her head and sit at eight months, could stand at one year and two months, and walk without help at two years of age. At the age of 12 years, she can say few monosyllabic words without an appropriate context [2]. In our case, the child was able to hold his head at two months, sit at seven months, and at the age of one year, he was able to stand and cruise around furniture by himself. At 25 months, he was able to say few words without forming a sentence. At the age of six years, he can say two to three words phrases and knows numbers and colors. Morán- Barroso et al. reported that pigmentary anomalies, like hyperpigmented and hypopigmented skin lesions, are present in 82% of patients with mosaic trisomy 13 [2]. No pigmentations or any type of skin lesions are present in the reported patient.

Our patient has typical dysmorphic features of trisomy 13 such as deep-seated eyes, small palpebral fissure, low set of ears, high arched palate, short neck, and right-hand polydactyly. Although cleft lip and palate are present in 80% of the cases, they were absent in our patient [9]. Microphthalmia and micrognathia are also absent. The observed cardiac malformations are patent foramen ovale, patent ductus arteriosus, and cardiomegaly which are consistent with the literature descriptions [9-10].

Only three osteomuscular abnormalities were present in our patient. Which included post-axial polydactyly in the right hand, bilateral leg intoeing, and internal tibial torsion. Other abnormalities like rocker-bottom feet, convex soles, and deep palmar creases were absent. Psychomotor retardation was noticed after the first year of life. Balance and gross motor issues improved with physiotherapy, and he is gaining new skills as he follows up with pediatric development clinics.

Management of patients with trisomy 13 should be tailored to the patient’s needs. Several factors should be considered when counseling the parents about the treatment options like parental autonomy, child’s best interest, and quality of life. Many patients will die within few weeks of life mainly due to central apnea, heart defects, and pulmonary hypertension. A better outcome is seen for trisomy 13 patients when receiving an intensive care treatment comparing to comfort care [6].

## Conclusions

Mosaicism of trisomy 13 is a rare genetic variant; it is reported to be present in 6% of patients with trisomy 13 and they are characterized by longer survival compared to Patau syndrome. We recommend early identification of central nervous system and cardiac malformations, and continuous blood glucose monitoring. We recommend early childhood intervention to help with the developmental delay. Parents should be educated about their child’s condition for better care, development, and growth.
